# A Case of Unilateral Coccidioidal Chorioretinitis in a Patient with HIV-Associated Meningoencephalitis

**DOI:** 10.1155/2019/1475628

**Published:** 2019-10-07

**Authors:** Christopher B. Toomey, Andrew Gross, Jeffrey Lee, Doran B. Spencer

**Affiliations:** ^1^Viterbi Department of Ophthalmology, Shiley Eye Institute, University of California at San Diego, San Diego, CA, USA; ^2^McGovern Medical School, University of Texas at Houston Health Science Center, Houston, TX, USA

## Abstract

Intraocular coccidioidomycosis is a rare condition, with the most commonly reported presentation being an idiopathic iritis in patients who live in or have traveled thorough endemic areas. A paucity of reports exists describing the chorioretinal manifestations of coccidioidomycosis. Here we report a case of unilateral coccidioidal chorioretinitis and meningoencephalitis in an AIDS patient that led to near complete unilateral loss of vision. A 48-year-old Hispanic female with poorly controlled HIV/AIDS in southern California presented with a three-week history of headache, nausea, vomiting, right eye blurry vision, and a one-day history of subjective fever. Examination of the right eye revealed vitritis and several large chorioretinal lesions scattered throughout the periphery and macula with optic disc pallor. Serum coccidioidomycoses complement fixation (CF) was positive (titers of 1 : 256). Neuroimaging revealed a new area of enhancement in the left anterior frontal lobe consistent with meningoencephalitis. The patient was treated with intravenous fluconazole and intravitreal voriconazole with resolution of systemic symptoms and vitritis but persistence of unilateral, severe chorioretinal scarring and vision loss. In conclusion, in spite of the rarity of intraocular coccidioidomycosis, one must carry a degree of suspicion for this vision- and life-threatening condition as a potential etiology of chorioretinitis in individuals with pertinent risk factors.

## 1. Introduction

Coccidioidomycosis is a disease caused most frequently by *Coccidioides immitis*, a dimorphic fungus commonly found in the lower Sonoran Desert ecozone of the Western hemisphere [1]. The disease, which typically manifests as a self-limiting community-acquired pneumonia, was initially described in the late 1800s and thought to be of protozoan etiology (and hence the etymology of Coccidia) but was later identified as a dimorphic fungus [2]. Endemic areas include Arizona, New Mexico, West Texas, parts of Central America, Argentina, Northwest Mexico, and the San Joaquin Valley in California [1]. Populations at greater risk include pregnant women and those with immunosuppressed conditions [3]. Symptomatic patients usually present with 1–3 weeks after exposure with flu-like symptoms including night sweats, cough, myalgias, and rash [4].

Uveitis secondary to coccidioidomycosis is rare, and the few reports of it most frequently describe an idiopathic, bilateral iritis in patients who live or have traveled thorough endemic areas [5, 6]. A paucity of reports exists describing the chorioretinal manifestations of coccidioidomycosis. We report an unusual, unilateral case of coccidioidal chorioretinitis with panuveitis and meningoencephalitis in a patient with AIDS that led to near complete, unilateral loss of vision.

## 2. Case Presentation

A 48-year-old Hispanic female presented to a HIV clinic in southern California with a three-week history of headache, right eye blurry vision, dizziness, myalgia, nausea, and vomiting, and a one-day history of subjective fever. The past medical history was significant for human immunodeficiency virus (HIV) for 13 years (CD4 103, viral load 877 two weeks prior) with chronic associated cytomegalovirus (CMV) viremia, mycobacterium avium-intracellulare infection and toxoplasmosis encephalitis. The patient's medications included abacavir, dolutegravir, and lamivudine, as well as azithromycin, ethambutol, pyrimethamine, sulfadiazine, and valganciclovir for opportunistic infections. Her temperature was 98.1°F, she had no abnormal vital signs, no recent weight change, no neurological symptoms and a normal systemic physical exam.

**Figure 1:**
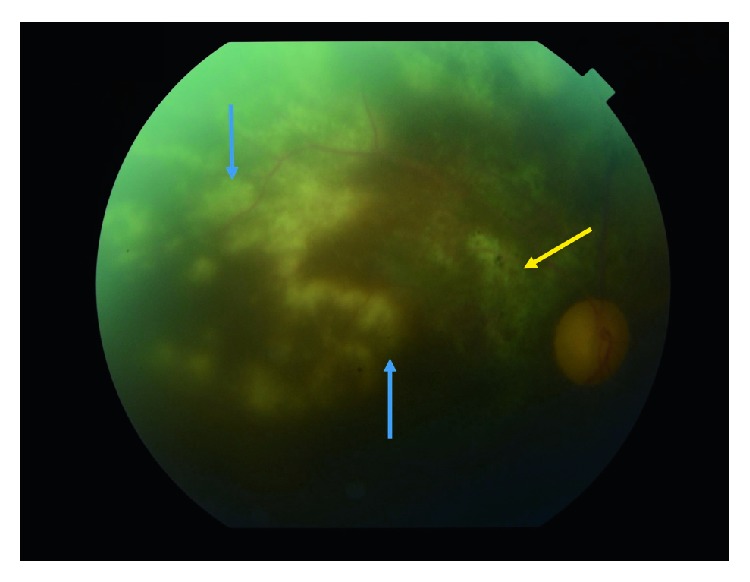
Fundus photograph with a yellow arrow marking an old, atrophic choroidal scar and blue lines delineating multiple white/off-yellow exudates around the macular and extending into the peripheral retina. Pallor of the optic disc is notable as well as minor vitreous haze.

Ophthalmic examination revealed visual acuity of hand motion at 1 foot in the right eye (OD) and 20/20 in the left eye (OS), pupil constriction of 3 > 3 mm OD and 3 > 2 mm OS, and a positive right-sided relative afferent pupillary defect (RAPD). Intraocular pressures were within normal limits and extraocular movements were full in both eyes. Confrontational visual fields and color plates were unable to be obtained OD and within normal limits OS. Anterior segment examination was within normal limits on bedside exam. Dilated fundus exam OD revealed 1+ vitreous cell/haze, several large chorioretinal lesions scattered throughout the periphery and involving the macula with 2+ optic disc pallor. OS was within normal limits (Figure 1).

**Figure 2:**
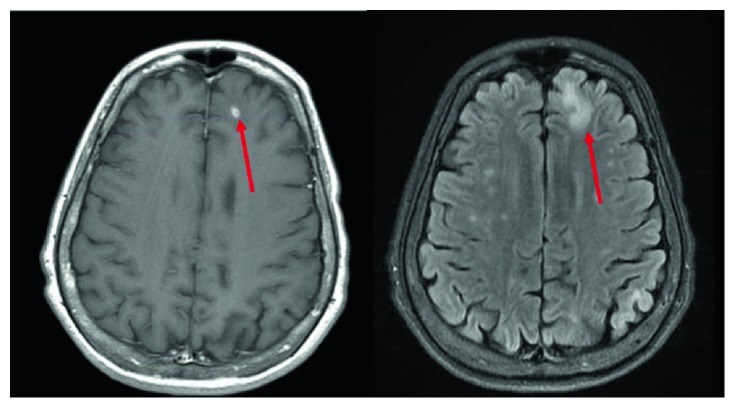
Axial T1 with contrast (left) and axial T2 FLAIR (right) magnetic resonance imaging (MRIs) demonstrating a peripherally enhancing 5 mm lesion in the left frontal lobe surrounded by moderate confluent vasogenic edema, marked by red arrows.

Due to the immunosuppressive state on presentation, as well as systemic symptoms, an extensive infectious work-up was performed as an inpatient with the Infectious Disease service and a vitreous tap was performed of the right eye. Serum coccidioidomycoses complement fixation (CF) was found to be positive (titers of 1 : 256). Serum syphilis enzyme immunoantibody, rapid plasma reagin, Borrelia burgdorferi antibody, CMV IgG/IgM, Varicella IgG, HSV type 1/2 IgM were negative. Gram stain showed polymorphonucleated white blood cells without organisms and cultures showed no growth. The encephalitis panel polymerase chain reaction was performed on the vitreous tap sample and was negative for Enterovirus, HSV1/2, Herpesvirus 6, Parechovirus, Varicella Zoster and *Cryptococcus neoformans/gatti*, E-coli K1, *Haemophilus influenza, Listeria monocytogenes, Neisseria meningitides, Strep. agalactiae, Strep. pneumoniae*. The vitreous PCR test was positive for Cytomegalovirus. CT head showed a new area of enhancement in the left anterior frontal lobe (Figure 2). Lumbar puncture (LP) with CSF gram stains, fungal cultures, and coccidioides CF were negative.

Based on the symptoms, positive serum coccidioidomycoses complement fixation and fundus appearance the patient was diagnosed with coccidioidal chorioretinitis with panuveitis. The vitreous PCR positive test was attributed to her long-standing CMV viremia secondary to sample contamination from the vitreous cells and trauma of the intravitreal biopsy and not deemed consistent with CMV retinitis given the absence of suspicious lesions. Additionally, her CD4 count of 103 places her outside of the highest risk strata of CMV retinitis, which occurs most commonly at a CD4 count of <50 [7]. The patient was treated with intravenous fluconazole and intravitreal voriconazole was preemptively given during the vitreous sampling. During her hospital course her systemic symptoms resolved. Follow-up examinations with serial fundus examinations showed progressive improvement in posterior vitritis, however, her visual acuity and retinal lesions remained stable throughout her hospital course. On follow-up examination at 3 and 5 months after discharge, her vision had decreased to light perception with no evidence of active lesions or vitritis and stable chorioretinal lesions.

## 3. Discussion

This case report describes the clinical manifestations of a patient with an unusual presentation of unilateral coccidioidal panuveitis with chorioretinitis; to our knowledge, this is unique in the literature. Few clinical reports exist describing the intraocular clinical manifestations of coccidioidomycosis [5, 6, 8–11]. Rodenbiker and Ganley reviewed a series of cases of ocular coccidioidomycoses with only 10 reported to have a posterior uveitis (retinitis, choroiditis, or chorioretinitis). The authors noted the anterior segment was often uninvolved and fundus findings included a range of lesions with yellow-white exudate ranging from <1 to 3 disc diameters [5]. Prevalence of asymptomatic chorioretinal scars in patients with coccidioidomycosis have been reported in 5 of 54 subjections (9.2%), suggesting the prevalence of coccidioidal chorioretinitis is higher than isolated reports suggest [12]. However, it is difficult to make assumptions on the prevalence of coccidioidal chorioretinitis in this manner given the difficulty of linking the chorioretinal scarring to the coccidioidomycosis.

The patient in this report presented with severe monocular vision loss with significant chorioretinal lesions and optic atrophy along with significant vitritis and a positive serum coccidioidomycoses CF. CSF coccidioidomycoses CF was negative, however, CSF testing has a low sensitivity in patients with coccidioidomycoses meningitis [13]. Our case report highlights the severity of coccidioidal chorioretinitis that can be associated with meningoencephalitis in a patient with HIV/AIDS, including in a unilateral fashion. Given the fundus appearance of old and new lesions, our clinical impression is that the chorioretinal involvement was on-going for many months and the patient presented for ophthalmologic evaluation once her central vision was affected. This supports the notion that ocular involvement of coccidioidomycosis may be an insidious process and highlights the essential role of routine ophthalmologic evaluation in AIDS patients in aiding in the diagnosis of systemic disease.

In spite of the rarity of intraocular coccidioidomycosis, recognizing the clinical appearance of this potentially vision and life-threatening condition is essential in individuals with pertinent risk factors including pregnancy and immunosuppression, along with a history of travel or living in endemic areas [1, 3].
